# Clinical and genomic determinants associated with emergent ceftazidime–avibactam plus aztreonam non-susceptibility in ceftazidime-avibactam resistant *Escherichia coli*

**DOI:** 10.1128/aac.01860-25

**Published:** 2026-03-23

**Authors:** William C. Shropshire, Jovan Borjan, Iris Y. Fonseca, Kathlyn Pham, Melanie Guerrero, Karl Liboro, Allyson Young, Jovelle K. Chung, Chin-Ting Wu, Ambrocio Manzanares, Micah M. Bhatti, Nancy N. Vuong, Guy Handley, Amy Spallone, Roy F. Chemaly, Ayesha Khan, Samuel Shelburne

**Affiliations:** 1 Department of Infectious Diseases, Infection Control, and Employee Health, The University of Texas MD Anderson Cancer Center 4002, Houston, Texas, USA; 2 Division of Pharmacy, The University of Texas MD Anderson Cancer Center 4002, Houston, Texas, USA; 3 Department of Pathology and Laboratory Medicine, School of Medicine, University of California Irvine 8788, Irvine, California, USA; 4 Department of Infection Control, Chief Quality Office, The University of Texas MD Anderson Cancer Center 4002, Houston, Texas, USA; 5 Department of Laboratory Medicine, The University of Texas MD Anderson Cancer Center 4002, Houston, Texas, USA; 6 Program in Diagnostic Genetics and Genomics, The University of Texas MD Anderson Cancer Center School of Health Professions 836304, Houston, Texas, USA; 7 Department of Genomic Medicine, The University of Texas MD Anderson Cancer Center 4002, Houston, Texas, USA; Entasis, Big Bay, Michigan, USA

**Keywords:** avibactam/aztreonam, ceftazidime/avibactam, metallo-beta-lactamases, plasmid mediated AmpC genes, novel beta-lactam/beta-lactamase inhibitors, ceftazidime/avibactam plus aztreonam

## Abstract

Ceftazidime–avibactam (CZA) has revolutionized care for carbapenem-resistant Enterobacterales infections, yet increasing New Delhi metallo-β-lactamase (NDM) prevalence has driven use of CZA plus aztreonam (ATM/CZA). We performed a comprehensive clinical and genomic analysis of ceftazidime–avibactam-resistant *Escherichia coli* (CZA-R-*Ec*) collected at a tertiary cancer center (2017–2024) to identify patient- and isolate-level factors associated with reduced susceptibility to ATM/CZA and aztreonam–avibactam (AZA). CZA-R-*Ec* were isolated from 48 unique patients of whom 28 (58%) had confirmed infection. Oxford Nanopore Technologies long-read sequencing performed on 34 isolates from unique patients showed a diverse population enriched for ST167 (35%). Most sequenced isolates carried *bla*_NDM-5_ (26/34, 76%); among *bla*_NDM-5_ strains, 88% (23/26) harbored PBP3 YRI(K/N) insertions. Eleven isolates (32%) carried *bla*_CMY_ variants, predominantly *bla*_CMY-42_. *Bla*_NDM-5_ and *bla*_CMY_ genes were largely plasmid-borne (IncF-type and IncI-γ/K1) in distinct genomic contexts. Among 32 confirmed CZA-R and ATM-R index isolates, 21 (66%) were ATM/CZA susceptible (MIC≤4 µg/mL; ATM/CZA-S). Compared to patients with ATM/CZA-S strains, patients with ATM/CZA non-susceptible (ATM/CZA-NS) isolates (ATM/CZA MIC>4 µg/mL) were significantly more likely to have had a prior *E. coli* infection (73% vs 0%, *P*-value = 1.6 × 10^−5^). ATM/CZA-NS isolates were strongly associated with *bla*_CMY_ carriage (81% vs 9% in ATM/CZA-S; adjusted *P*-value = 4 × 10^−4^). Among 18 confirmed CZA-R-*Ec* bacteremias, 15 carried *bla*_NDM_; 14/15 (92%) received ATM/CZA and all responded clinically. In conclusion, CZA-R-*Ec* at our center are dominated by PBP3-insertion, *bla*_NDM-5_–positive lineages, for which ATM/CZA retains substantial *in vitro* activity and clinical efficacy. However, prior *E. coli* infection and *bla*_CMY-42_ variant positivity identify patients at risk for ATM/CZA non-susceptible infections.

## INTRODUCTION

The introduction of ceftazidime/avibactam (CZA) along with other novel β-lactam/β-lactamase inhibitor combinations (BL/BLIs) has offered improved treatment options for carbapenem-resistant Enterobacterales (CRE) infections (1–3). Avibactam broadly inhibits Class A, C, and D β-lactamases, restoring ceftazidime activity against diverse Gram-negative organisms, including carbapenemase producers (1, 2). However, avibactam does not inhibit metallo-β-lactamases (MBL) such as the New Delhi metallo-β-lactamase (NDM).

Therefore, in the context of MBL positive infections, clinicians have utilized CZA in combination with aztreonam (ATM), which relies on the concept that ATM is not hydrolyzed by MBLs while avibactam can inhibit many non-MBL ATM hydrolyzing enzymes (e.g*.*, *bla*_CTX-M_, *bla*_CMY_, and *bla*_OXA_) often co-carried in MBL producing strains. *In vitro* data support the synergistic efficacy of this combination (4), with fractional inhibitory concentration indices (FICI) <0.5 reported against various carbapenemase-producing strains (5, 6). While large randomized controlled trials have evaluated the efficacy of CZA in treating Gram-negative infections broadly (7, 8), clinical data focused on MBL-producing *E. coli* and their association with ceftazidime-avibactam plus aztreonam (ATM/CZA) remain limited; in particular, most available evidence comes from case reports or small case series documenting microbiological eradication following ATM/CZA therapy (9–12). Moreover, there are limited studies on the clinical effectiveness of ATM/CZA, particularly in immunocompromised hosts (11).

Notably, the *bla*_NDM-5_ MBL gene variant is being detected in multi-drug resistant (MDR) *E. coli* infections with increasing frequency worldwide (9, 13–19). Whole genome sequencing (WGS) studies have identified several high-risk, MDR *E. coli* multilocus sequence types (MLSTs) associated with *bla*_NDM-5_ carriage, particularly ST167, ST101, ST410, ST361, and ST405, with a varied, worldwide geographic distribution (13–15, 17–20). Many of these high-risk lineages possess fixed mutations in *ftsI*, encoding penicillin-binding protein 3 (PBP3), a principal β-lactam target, resulting in reduced affinity for agents such as ATM (17, 19, 21, 22). These PBP3 mutations, in particular stable, four amino acid insertions of YRIN or YRIK following residue P333, have been associated with compromised *in vitro* ATM/CZA activity in clinical NDM-producing pathogens, which further complicates treatment modalities (19, 20, 22, 23). Simner et al. reported a case in which an insertion sequence (i.e., IS*26*) based pseudocompound transposon (PCTN) was associated with increased copy number and expression of the *bla*_NDM-5_ gene with corresponding elevations in ATM/CZA and cefiderocol (FDC) minimum inhibitory concentrations (MICs) showing how gene dosage in the context of these *ftsI* insertion backgrounds can lead to pan-resistant infections (24). Furthermore, we and others have recently detected high-risk *E. coli* lineages harboring plasmid mediated AmpC genes, in particular *bla*_CMY-2_ variants often in the presence of these *ftsI* mutations, which have high CZA MICs as well as reduced ATM/CZA activity (25–28).

While the aforementioned case reports or genomic analyses have provided important insights, there is a paucity of studies dissecting resistance mechanisms by characterizing the relationship between genotypic, phenotypic, and clinical outcome data. To address this gap, we performed a retrospective analysis of patients with CZA resistant *E. coli* (CZA-R-*Ec*) isolated in culture at a tertiary care cancer center between 2017 and 2024. We aimed to elucidate molecular mechanisms driving reduced susceptibility to ATM/CZA or aztreonam-avibactam (AZA) and subsequent impact on clinical outcomes by integrating patient-level data with comprehensive whole genome sequencing analysis and antimicrobial susceptibility testing.

## RESULTS

### There has been a gradual increase in absolute frequency of annual CZA-R-*Ec* infections at our institution

Between 2017 and 2024, CZA-R-*Ec* were cultured from 48 unique patients as part of routine clinical care at The University of Texas MD Anderson Cancer Center in Houston, Texas. Table 1 provides a descriptive overview of these patients. The absolute frequency of CZA-R-*Ec* isolated from patients was 18 cases detected between 2017 and 2020 compared to 30 cases from 2021 to 2024 (Fig. S1). Many patients (*n* = 23) were neutropenic (ANC < 500 cells/mm^3^) at the time of index CZA-R-*Ec* isolation, and 27% (13/48) were hematopoietic stem cell transplant (HSCT) recipients. A total of 58% (28/48) CZA-R-*Ec* cases were confirmed infections with the majority being bloodstream infections (68%, *n* = 19). The most common treatment modality was ATM/CZA (74%; 20/27) with one patient mortality occurring prior to initiation of treatment. The overall 30-day mortality following index CZA-R-*Ec* collection was 13% (6/48), with four deaths occurring in patients with confirmed CZA-R-*Ec* infection.

**TABLE 1:** Clinical characteristics of 48 patients from which CZA-R-*Ec* were isolated

Covariate	*N*, % unless specified
Age, median (min, max)	63 (18, 88)
Gender, male	28 (58)
International travel in previous 12 months	25 (52)
Neutrophil count < 500 cells/mm3	23 (48)
Hematopoietic Stem Cell Transplant Recipient	13 (27)
Infection type (*n* = 28)	
Blood	19 (68)
Urine	3 (11)
Sputum	2 (7)
Other	4 (14)
Treatment (*n* = 27)	
ATM/CZA	20 (74)
FDC	3 (11)
Other	4 (14)
30-day mortality	6 (13)

### CZA-R-*Ec* primarily consist of high-risk lineages harboring *bla*_NDM-5_ and *ftsI* insertions

**Fig 1:**
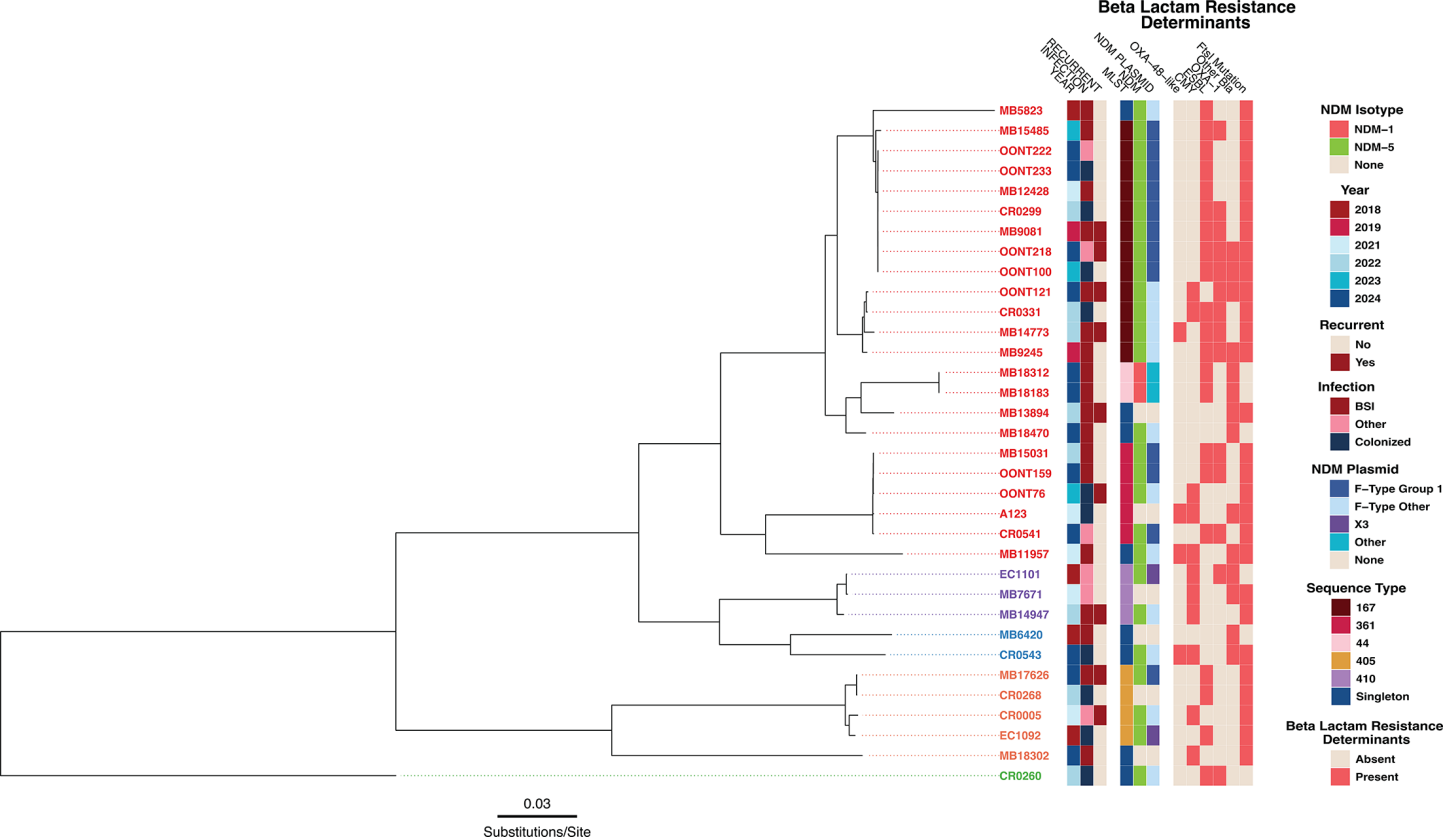
Core genome alignment maximum-likelihood inferred phylogeny of first occurrent CZA-R-*Ec* isolates (*N* = 34) collected from 2018 to 2024. Terminal branch tips are colored based off phylogroup with phylogroup A (red), C (purple), B1 (blue), D (orange), and B2 (green) observed from top to bottom. Metadata from the heatmap is labeled accordingly in the legend present to the right of the phylogeny.

There were 34 isolates from unique patients available for analysis. A full list of genomic data for these plus recurrent isolates (*n* = 7) sequenced can be found in Table S1. Clinical data comparison from patients with sequenced (*n* = 34) vs non-sequenced (*n* = 14) isolates (Fig. S1) showed that sequenced isolates were more likely to have been infections (71% vs 36%; *P*-value = 0.05) and isolated from blood (53% vs 14%; *P*-value = 0.03), but there was no significant difference among phenotypic carbapenemase testing results (80% vs 86%; *P*-value: 1). Fig. 1 provides an overview of the population structure as well as genomic features associated with CZA-R-*Ec* collected isolates. The most commonly detected sequence type (ST) was ST167 (35%; *n* = 12), with other high-risk lineages such as ST361 (15%; *n* = 5), ST405 (9%, *n* = 4), ST410 (6%, *n* = 3), and ST44 (6%, *n* = 2) being observed more than once. Using a core genome, recombination masked alignment, we identified two clusters of closely related CZA-R-*Ec* isolates (i.e., single nucleotide polymorphism [SNP] pairwise distances < 15 [29]) labeled Group 1 (ST167; *n* = 5) and Group 2 (ST44; *n* = 2), respectively (Fig. S2, Table S1), suggesting potential transmission despite any clear hospital associated epidemiological links observed by our infection control team. Group 1 consisted of *bla*_NDM-5_ positive ST167 isolates harboring p.P333insYRIN *ftsI* mutations, whereas Group 2 consisted of two *bla*_NDM-1_ positive ST44 isolates. Pairwise SNP distances for these isolates can be found in Table S2.

The majority (76%; 26/34) of the CZA-R-*Ec* isolates were *bla*_NDM-5_ positive with only two isolates (MB18183 and MB18312) harboring *bla*_NDM-1_ genes. Most of the *bla*_NDM-5_ positive isolates (88%; 23/26) had an *ftsI* mutation (17 with p.P333insYRIN and 6 with p.P333insYRIK). With the exception of one MBL positive isolate (MB18470), all MBL positive strains carried a combination of *bla*_CTX-M_ (*bla*_CTX-M-15_ [*n* = 13], *bla*_CTX-M-14_ [*n* = 2], *bla*_CTX-M-14_/ *bla*_CTX-M-15_ [*n* = 1]), *bla*_OXA-1_ (*n* = 14), *bla*_CMY_ (*bla*_CMY-42_ [*n* = 5], *bla*_CMY-4_ [*n* = 1], *bla*_CMY-59_ [*n* = 1], *bla*_CMY-145_ [*n* = 1]), and/or *bla*_OXA-48-like_ (*bla*_OXA-48_ [*n* = 1], *bla*_OXA-1181_ [*n* = 1], *bla*_OXA-1207_ [*n* = 1]) genes, which further highlights the MDR genotype of these pathogens (Table S1). *Bla*_CMY-42_ variants were present in three of the six strains that were *bla*_NDM_ negative (Fig. 1).

### Integration of clinical and genomic data for CZA-R-*Ec* causing bloodstream infections

To integrate clinical and genomic data, we focused on the 18 patients with CZA-R*-Ec* bloodstream infections with sequencing data available, all of whom had hematologic malignancies and an absolute neutrophil count (ANC) < 500 cells/mm^3^. *Bla*_NDM_ was present in 15 of the 18 isolates. Among the three patients infected with MBL-negative strains, two died within 30 days. The third patient with an MBL-negative strain died 60 days after receiving sequential therapy with FDC, ATM/CZA plus tigecycline, and meropenem–vaborbactam plus eravacycline. Of the 15 patients with *bla*_NDM_ isolates, all but one were treated with ATM/CZA and had a favorable clinical response. The remaining one patient was treated with meropenem-vaborbactam since the isolated tested susceptible in the clinical laboratory and clinically improved*.* The median time to effective therapy was 45 h (minimum 1, maximum 115), and the median time to clinical response after starting effective therapy was 23 h (minimum 9, maximum 117). Based on *in vitro* susceptibility data, eight patients received effective antibiotics in addition to β-lactams (seven received tigecycline and one received amikacin). Median duration of effective treatment was 15 days (minimum 7, maximum 34). Four of the 15 patients with *bla*_NDM_-positive bloodstream infections experienced recurrent bacteremia, and three of these patients were successfully re-treated with β-lactam/β-lactamase inhibitor plus ATM combinations.

### ATM/CZA *in vitro* synergy was observed in the majority of CZA-R-*Ec* isolates

Antimicrobial susceptibility (AST) results are shown in Table S3 for the 34 index and 7 recurrent isolates that tested initially CZA-R based off electronic health records. We confirmed CZA-R phenotypes in all but one of our 34 available isolates (MB6420), which tested CZA-S upon repeat testing (Table S3). This index bloodstream infection isolate has truncated *ompC* and *ompF* genes with concomitant increased *bla*_TEM-1_ gene copy numbers (~150 copies); these gene copy numbers can be unstable and could account for the loss of CZA-R phenotype (29–32). We further excluded an additional isolate that tested CZA-R but ATM-S (MB18470) from our ATM/CZA analysis with this phenotype likely due to the absence of β-lactam resistance mechanisms other than *bla*_NDM-5_ and *bla*_TEM-1_.

**Fig 2:**
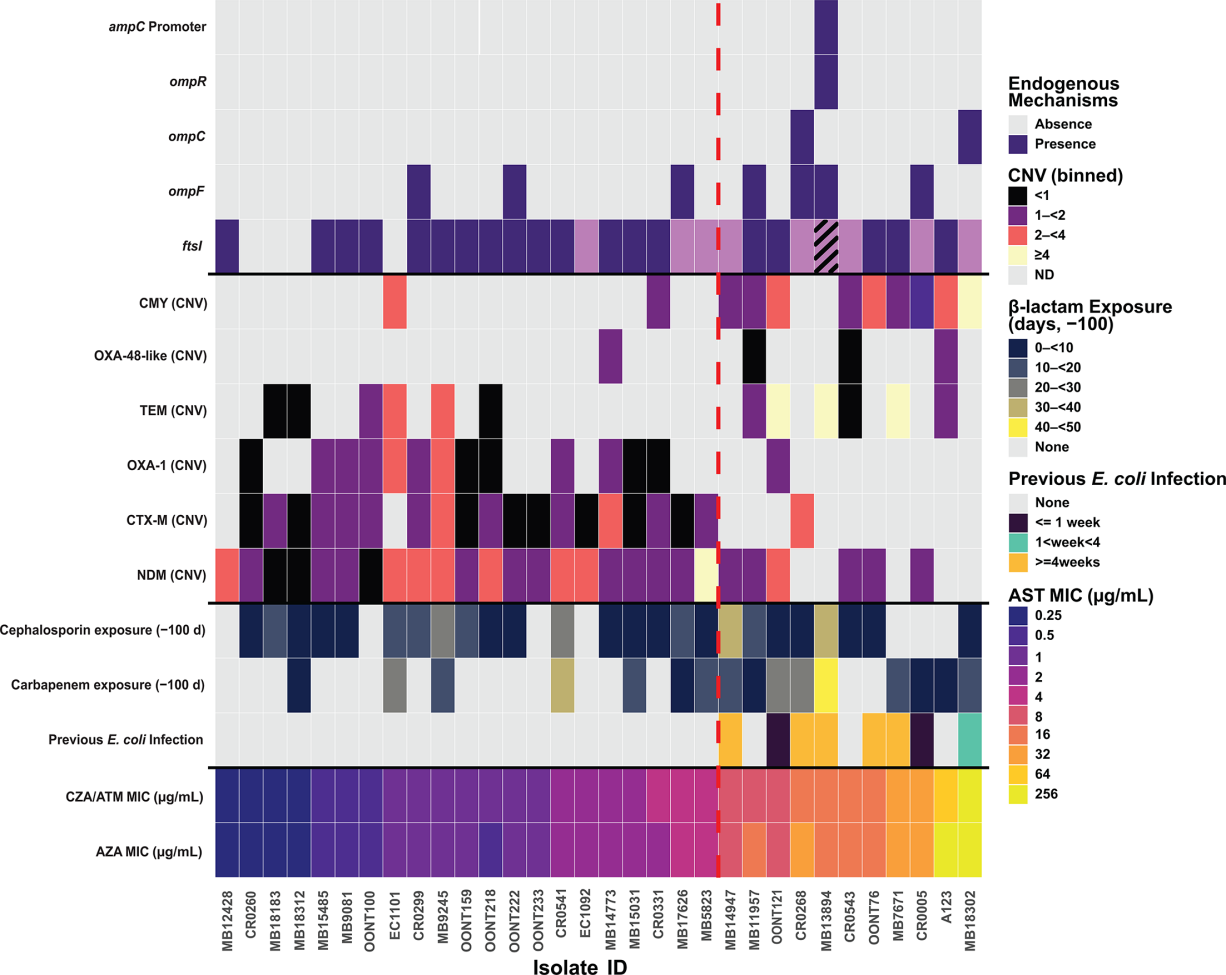
Clinical and genomic features associated with increasing ATM/CZA and AZA MICs. The *X*-axis is sorted first by increasing ATM/CZA MIC and then by increasing ATM and CZA MIC, respectively. *Y*-axis from top to bottom indicates: Rows 1–5: presence/absence of chromosomal mutations predicted to impact β-lactam resistance; Rows 6–11: presence/absence and copy number variation (CNV) of exogenous B-lactamase content; Rows 12–14: Clinical metadata. Vertical dotted red line indicates the cutoff between ATM/CZA-S (left) and ATM/CZA-NS (right). Metadata from the heatmap is labeled accordingly in the legend present to the right of the figure. Note for *ftsI* mutations, dark purple indicates predicted YRIN mutation, whereas light purple indicates predicted YRIK mutation. The striped *ftsI* rectangle indicates a point mutation (p.A498T) associated with cephalosporin resistance as annotated in AMRFinderPlus (33).

Figure 2 provides clinical and genomic details for the first available CZA-R and aztreonam resistant (ATM-R) isolates upon which we performed ATM/CZA MIC testing. Among 32 confirmed CZA-R and ATM-R-*Ec* index isolates tested with reference broth microdilution, 21 (66%) had ATM/CZA MICs <=4 µg/mL, which we define as ATM/CZA susceptible (ATM/CZA-S) based off EUCAST guidelines (30). Of the 11 ATM/CZA non-susceptible isolates (ATM/CZA MICs >4 µg/mL; ATM/CZA-NS), eight (73%) were from patients who had at least one *E. coli* infection in the 100 days prior to the index CZA-R-*Ec* isolate (Fig. 2; Table 2), whereas none of the patients with ATM/CZA-S isolates had a previous *E. coli* infection within this timeframe (*P*-value = 1.6 × 10^−5^). A comparable proportion of patients had previous cephalosporin exposure within 100 days from index CZA-R-*Ec* isolate for those with ATM/CZA-NS (8/11; 73%) vs ATM/CZA-S (17/21; 81%; adjusted *P*-value = 0.3) strains. However, a higher proportion of patients with ATM/CZA-NS isolates had previous carbapenem exposure in the 100 days prior to CZA-R-*Ec* isolation (9/11; 82%) compared to ATM/CZA-S isolates (7/21; 33%) (Table 2; adjusted *P*-value = 0.05). The median days of any cephalosporin exposure 100 days prior in the ATM/CZA-S group (6 days; Interquartile range [IQR]: 9 days) were similar in duration to the ATM/CZA-NS group (4 days; IQR: 7 days) (adjusted *P*-value = 0.4). The median days of any carbapenem exposure in the 100 days prior were significantly greater in the ATM/CZA-NS group (12 days; IQR: 13 days) compared to the ATM/CZA-S group (0 days; IQR: 7 days) (adjusted *P*-value = 0.02).

**TABLE 2:** Comparison of ceftazidime/avibactam + aztreonam ATM/CZA-S vs ATM/CZA-NS strains^*a*^

Covariate	ATM/CZA-S (*n* = 21)	ATM/CZA-NS (*n* = 11)	*P*-value^*b*^
Prior *E. coli* infection	0 (0)	8 (73)	1.6e−5
Cephalosporin receipt within prior 100 days	17 (81)	8 (73)	0.3
Days of cephalosporin therapy prior 100 days, median (IQR)	6 (9)	4 (7)	0.4
Carbapenem receipt within prior 100 days	7 (33)	9 (82)	0.05
Days of carbapenem therapy prior 100 days, median (IQR)	0 (7)	12 (13)	0.02
*Bla*_CMY_ positivity	2 (10)	9 (82)	4.1e−4
*Bla*_NDM_ positivity	21 (100)	6 (55)	6.9e−3
*Bla*_ESBL_ positivity	19 (91)	1 (9)	9.3e−5
*ftsI* mutation	17 (81)	11 (100)	0.3
*ompC* and/or *ompF* mutation	3 (14)	6 (55)	0.04

^*a*^ IQR, interquartile region.

^*b*^ *P*-value calculated using the Fisher’s exact test.

A notable observation was the higher proportion of ATM/CZA-NS isolates that carried *bla*_CMY_ (9/11; 82%) in contrast to ATM/CZA-S isolates (2/23; 10%) (adjusted *P*-value: 4.1 × 10^−4^) (Fig. 2; Table 2). Further stratification of ATM/CZA MICs by *bla*_NDM_ and *bla*_CMY_ carriage shows there is a statistically significant higher median ATM/CZA MIC in *bla*_NDM_+/*bla*_CMY_+ isolates (8 μg/mL, IQR:9) compared to *bla*_NDM_+/*bla*_CMY_- isolates (1 μg/mL, IQR:1.5) (Fig. 2; Fig. S3). Comparing *bla*_CMY_ variants by ATM/CZA susceptibility status (i.e., CMY alleles with or without p.V221 mutations relative to CMY-2), all ATM/CZA-NS *bla*_CMY_+ isolates had a p.V221 mutation with two isolates also harboring p.N346 mutations (MB7671 and A123). *Bla_CMY_* variants detected by ATM/CZA susceptibility with additional genomic context data (e.g., other *bla* genes, copy numbers) can be found in Table S4. MB7671 has been previously described (25, 26) as having four mutations relative to CMY-2 (Ambler positions A114E, Q120K, V211S, N346Y). Kawai et al*.* established p.N346Y as the main driver of CMY-185-mediated CZA resistance by both blocking avibactam binding and enhancing ceftazidime hydrolysis, while V211S further boosts resistance by increasing the turnover rate of oxyimino-cephalosporin hydrolysis (26). Strain A123 carries a novel *bla*_CMY_ gene, designated *bla*_CMY-225_, which has three mutations relative to CMY-2 (Ambler positions N70T, V211S, and N346D) and a concomitantly high ATM/CZA MIC (64 μg/mL; Fig. 2) in a PBP3 p.P333insYRIN background. The two ATM/CZA-S strains which encoded *bla*_CMY_ included one *bla*_CMY-42_ isolate with a wild type *ftsI* background (EC1101) and another (CR0331) which contained *bla*_CMY-4_ (*i.e.*, non *bla*_CMY-42-like_) variant.

Figure 2 and Table 2 also highlight additional genomic features such as PBP3 predicted YRI(K/N) duplication mutations being present in all ATM/CZA-NS isolates and most ATM/CZA-S (17/21, 81%) isolates (adjusted *P*-value = 0.3). There was a statistically significant higher ATM/CZA MIC in PBP3 YRI(K/N) positive isolates (3 μg/mL; IQR: 15 μg/mL) compared to PBP3 YRI(K/N) negative isolates (0.25 μg/mL; IQR: 0.19 μg/mL) (adjusted *P*-value = 0.02). We further found that PBP3 YRIK+ isolates (*n* = 8) had a statistically higher ATM/CZA MIC (median 12 μg/mL; IQR: 16) compared to PBP3 YRIN+ isolates (*n*=19) (median 1 μg/mL; IQR: 15) (pairwise Mann-Whitney *P*-value: 0.04). Carriage of *bla*_NDM_, *bla*_CTX-M_, and *bla*_OXA-1_ was more common in ATM/CZA-S isolates (100%, 91%, and 62%, respectively) compared to ATM/CZA-NS isolates (55%, 9%, and 9%, respectively) (Table 2; adjusted *P*-values < 0.02). The two ATM/CZA-NS/*bla*_CMY_- isolates lacked *bla*_NDM_ as well and instead carried disparate genomic backgrounds. One isolate (CR0268) was *ompC/ompF*-deficient and carried a predicted PBP3 p.P333insYRIK mutation with *bla*_CTX-M-15_. The other (MB13894) harbored *ompF* and *ompR* disrupting mutations along with *ampC* promoter mutations in addition to *bla*_TEM-1_. When testing differences in copy number variants (CNVs) of *bla*_NDM-5_, there was no discernible difference in ATM/CZA-S (mean 1.9×; standard deviation [SD]: 1.2) and ATM/CZA-NS (mean 1.6×; SD: 0.36; *P*-value = 0.7) strains.

Finally, we compared the distribution of ATM/CZA with aztreonam-avibactam (AZA) MICs (Fig. 2; Table S3). With the exception of one isolate (A123; ATM/CZA MIC = 64 µg/mL; AZA MIC = 256 µg/mL), all were in essential agreement (i.e*.*, within one doubling dilution; Fig. 2; Table S3), with no statistically significant difference in MIC distributions (Wilcoxon on log2 MICs: *P*-value = 0.2). Furthermore, when comparing results from ATM/CZA reference broth microdilution and the qualitative ETEST-based strip cross method (31), we found perfect concordance when calling ATM/CZA MICs <=4 µg/mL vs >4 µg/mL, reaffirming the practicality and reliability of the latter method to detect ATM/CZA-NS CZA-R*-Ec* strains in the clinical setting (Table S3).

### *Bla*_NDM-5_ and *bla*_CMY_ variants predominantly carried on highly conserved F-type and IncI-γ/K1 plasmids

With the exception of a single patient (CR0005/CR0063 isolates) in whom *bla*_CMY_ occurred in a chromosomal rather than plasmid context, all *bla*_CMY_ and *bla*_NDM_ genes were plasmid-borne. In addition, nearly all confirmed CZA-R-*Ec* isolates (30/32) harbored *bla*_NDM_ and/or *bla*_CMY_; thus, we performed a comprehensive comparative plasmid analysis to (i) determine how these AMR genes clustered with previously characterized complete plasmids available on NCBI, (ii) assess whether β-lactamase gene copy number was driven by plasmid copy number or by mobile genetic elements (MGEs), and (iii) identify the associated mobilization mechanisms.

**Fig 3:**
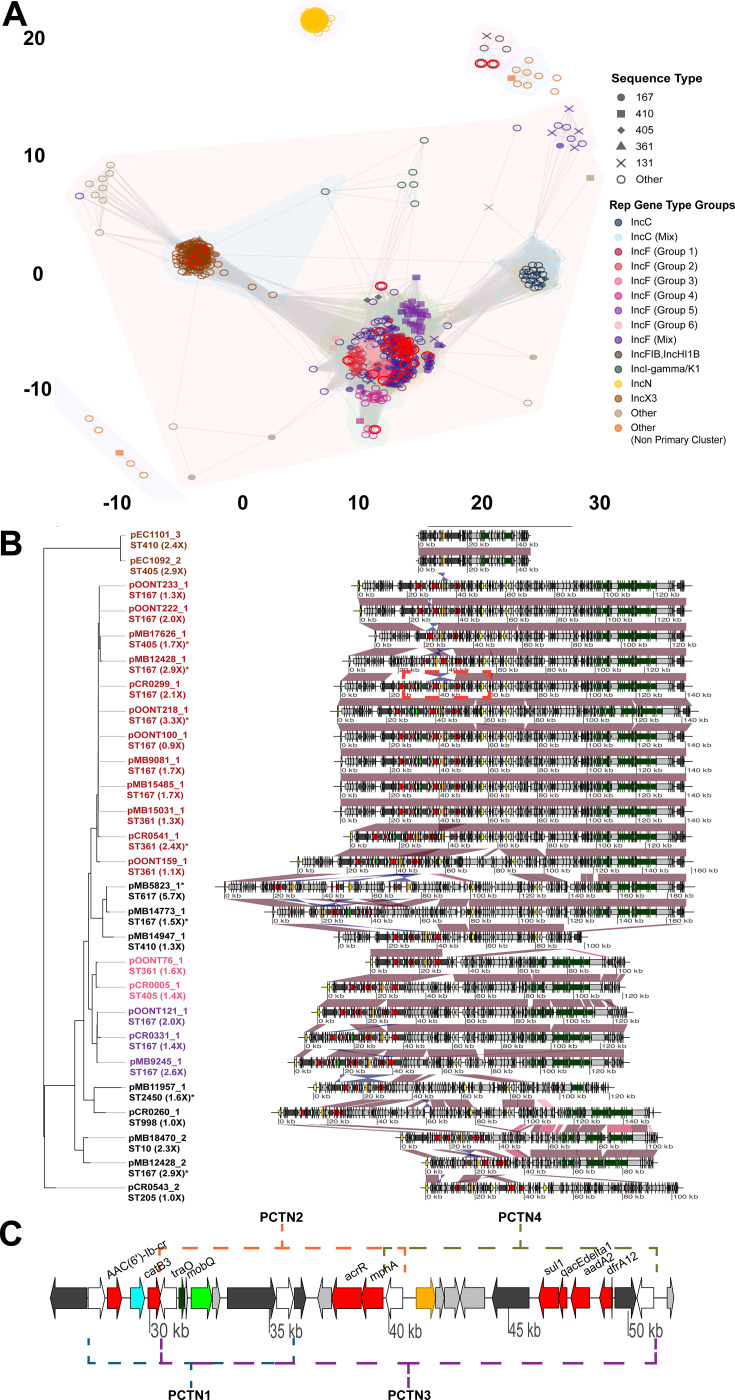
Plasmids harboring *bla*_NDM_ comparative genomics analysis. (**A**) Network analysis of full-length plasmids harboring *bla*_NDM-1_ and *bla*_NDM-5_ identified in our study (red circles around shapes) and NCBI data abstracted using Pathogen Detection. Shapes indicate most commonly identified sequence types and color represent Inc type groupings based off Pling clustering results. Subcommunity linkage is indicated by gray edges connecting nodes (i.e., plasmid sequences). Peripheral groups (e.g., IncN plasmids) share no linkage with primary community identified in the center of the figure. (**B**) Blastn comparison of full-length multireplicon plasmids. Branch tips are colored by groupings established in subcommunity analysis with sequence type labeled. Black font indicates “singleton” plasmids that don’t group with another plasmid in our cohort. Number in parentheses represents estimated *bla*_NDM-5_ copy number from AMR-STRUCT with asterisk indicating increase in *bla*_NDM-5_ copy number was associated with PCTN amplification. Red and blue shading between sequences indicates >95% shared blastn ID in direct and reverse orientation respectively. *Bla*_NDM-5_ (orange), *bla*_CTX-M-15_ (green), *bla*_OXA-1_ (blue), other AMR genes (red), MGE content (dark gray), rep genes (yellow), IS6 family transposase gene (white), Tn3 genes (brown), and conjugal transfer genes (dark green) are labeled accordingly. Plasmids reoriented to IncF replication initiation gene (yellow) for DNA comparisons. Dotted region indicates zoomed region in the following subsection. (**C**) Zoomed- in region of F-Type plasmid (Group 1; 25,844–51,913 bp—pCR0299_1 reference) with well conserved PCTNs detected in Group 1 plasmids.

Figure 3A shows that most *bla*_NDM-5/-1_ plasmids in our combined cohort and public data set cluster within IncF subcommunities; IncF subtypes predominantly carry *bla*_NDM-5_, whereas *bla*_NDM-1_ is chiefly associated with IncC and IncN subcommunities (Fig. S4A). Similar to our strains, we found that 20% of *bla*_NDM_ positive plasmids originated from ST167 strains (147/724) with many of the IncF *bla*_NDM-5_ positive isolates having been collected in the USA and Europe in the past four years (Fig. S4B and C, Table S5).

Focusing on our *bla*_NDM-5_ plasmids, nearly half (12/27) belonged to a predicted conjugative IncFIA, IncFIB, IncFIC, IncFII multireplicon plasmid cluster (IncF Group 1) which shares megablast weighted averages of >99% identity and 95% coverage (Fig. 3B). The cargo gene region for this plasmid cluster includes a well conserved, mosaic ~25 kb pseudocompound transposon (PCTN) that carries *bla*_NDM-5_ in addition to *bla*_CTX-M-15_ and *bla*_OXA-1_ with four predicted IS*26* PCTN units (Fig. 3C) based on previous studies of IS*26* mobilization mechanisms (32). Using AMR-STRUCT (see Materials and Methods), 4 of the 12 IncF Group 1 plasmids had predicted PCTN increased copies based off increase in coverage depths within PCTN region relative to the total plasmid coverage depth or 2 or more resolved copies in the complete assembly (Fig. 3C). Three amplified units are PCTN4 detected on pCR0541_1, pOONT218_1, and pMB17626_1 with two copies of PCTN4 fully resolved on pCR0541_1 (Fig. 2B). The PCTN harboring *bla*_NDM-5_ on pMB12428_1 is a novel 15.8kb PCTN that has an additional copy on pMB12428_2 present on a unique IncFIA,IncFIC 92.2kb plasmid.

**Fig 4:**
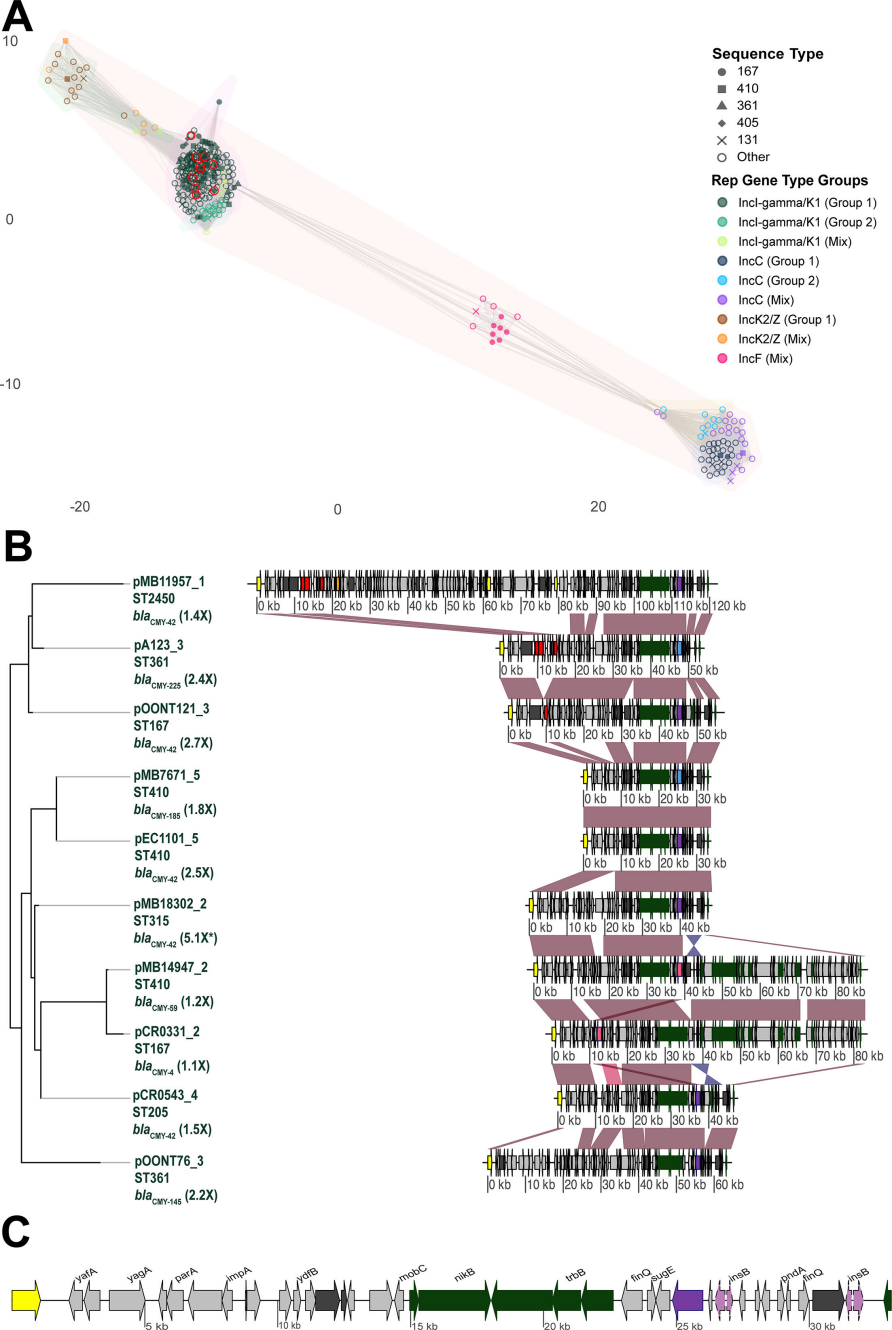
Plasmids harboring *bla*_CMY_ comparative genomics analysis. (**A**) Network analysis of full-length plasmids harboring *bla*_CMY-2_ and *bla*_CMY-2_ variants identified in our study (red circles around shapes) and NCBI data abstracted using Pathogen Detection. Shapes indicate most commonly identified sequence types and color represents Inc type groupings based off Pling clustering results. Subcommunity linkage is indicated by gray edges connecting nodes (i.e., plasmid sequences). (**B**) Blastn comparison of full-length IncI-γ/K1 plasmids. All plasmids belonged to group 1 as represented in (**A**). Number in parentheses represents estimated *bla*_CMY_ copy number from AMR-STRUCT with asterisk indicating increase in *bla*_CMY_ copy number due to plasmid and transposon amplification. Red and blue shading between sequences indicates >95% shared blastn ID in direct and reverse orientation, respectively. *Bla*_NDM-5_ (orange), other AMR genes (red), MGE content (dark gray), rep genes (yellow), IS6 family transposase gene (white), Tn3 genes (brown), and conjugal transfer genes (dark green) are labeled accordingly. Non-V211S blaCMY-2 variants are pink, V211S *bla*_CMY-2_ variants are labeled in purple, and V211S *bla*_CMY-2_ variants with p.N346 mutations are labeled in blue. (**C**) Representative IncI-γ/K1 plasmid (pEC1101 reference) with IS1R genes upstream labeled in light pink and dotted outline stroke.

Figure 4A provides an overview of the network of *bla*_CMY_ positive plasmids abstracted from NCBI in addition to those from our own cohort. The majority of *bla*_CMY_ positive plasmids (*n* = 310) belonged to IncI-γ/K1 based subcommunities (71%; 219/310) with two other groups (IncC/IncK2 based subcommunities) also present (Fig. 4A). When stratifying the IncI-γ/K1 subcommunities by *bla*_CMY_ variant, Group 1 carried predominantly *bla*_CMY-42_ or *bla*_CMY-42_ like variants (70%; 132/188), in particular, variants with the V211S amino acid substitution, whereas Group 2 carried *bla*_CMY-2_ (96%; 22/23) (Fig. 4A; Fig. S5).

All 10 of the *bla*_CMY_ plasmids in our cohort belonged to the Group 1 IncI-γ/K1 subcommunity which shared a mean 98.6% megablast identity and 71% coverage (Fig. 4B). Seven of the 10 plasmids had *bla*_CMY_ estimated copy numbers >1.5× with all seven estimated to be due primarily to plasmid copy number changes (Fig. 4B). Figure 4C illustrates a representative IncI-γ/K1 plasmid (pEC1101_5) with the highly conserved gene order of transposase IS1R (*insB*) upstream of *bla*_CMY_ shaded in purple/blue dependent on *bla*_CMY_ variant type (Fig. 4B). Interestingly, pMB7671_5 and pEC1101_5 shared >99.9% blast identity, 100% coverage and estimated plasmid copy number >1.5× , with the notable difference that pMB7671_5 harbored the inhibitor resistant *bla*_CMY-185_ variant whereas pEC1101_5 harbored *bla*_CMY-42_ (Fig. 4B). *Bla*_NDM_ and *bla*_CMY_ were almost always on distinct plasmids, with only one plasmid (pMB11957_1) co-carried *bla*_NDM-5_ and *bla*_CMY-42_ in our cohort (Fig. 4B), which was a recombined IncI-γ/K1 and IncFIB/IncFII plasmid (Table S6). This low plasmid co-carriage was consistent with our global CMY plasmid analysis where only 2% (5/310) of *bla*_CMY_ positive plasmids co-carried *bla*_NDM-5_ (Fig. S5, Table S6).

### Recurrent CZA-R-*Ec* isolates share similar ATM/CZA phenotypes as index isolates

We had seven patients with index CZA-R-*Ec* and recurrent CZA-R-*Ec* isolates available for sequencing (Table S7). The median time from index to recurrent isolate collection for these pairs was 113 days (range: 49–569 days). All paired isolates were collected from infections (5 bloodstream, 2 other), and the median number of recurrences was 2 (range: 1–5). Two patients (Patient 2 and Patient 5) died within 25 and 6 days, respectively, of their final recurrent CZA-R-*Ec* bacteremia. When performing comparative genomics on index to recurrent isolate, the median pairwise SNP distance was 3 (range: 2–6) (Table S7).

Among the seven patients with paired index and recurrent CZA-R-*Ec* isolates, two pairs were classified as ATM/CZA-NS with the remaining five patients having ATM/CZA-S paired isolates (Table S7). The only sequential isolates with a substantial ATM/CZA and/or AZA MIC change (i.e., ±one doubling MIC dilution for recurrent isolate) were from patient 2 (pairwise SNP distance: 6), which were also the only *bla*_NDM-5_ negative recurrent isolates. Genomic changes for these isolates included a predicted p.Leu369Phe mutation in PBP3 (i.e., c.1107A>T in *ftsI*), as well as mutations in the efflux RND transporter permease AcrB (p.Phe136Leu) and envelope stress sensor histidine kinase CpxA (p.Gln120Lys) (Table S8). We also observed a *bla*_NDM-5_, *bla*_OXA-1_, and *bla*_CTX-M-15_ CNV increase in Patient 5 from 1.5× to 4.5× (Fig. S6) which was found to map to a 20 kb unique PCTN as presented in Fig. 3B and Fig. S6**;** however, there was only a twofold increase in ATM/CZA and AZA MICs (Table S2).

## DISCUSSION

Over the past 15 years, most advances in β-lactam therapy of drug-resistant Gram-negative bacteria have centered on new BL/BLI combination treatments. Novel agents such as CZA have improved outcomes, particularly for infections caused by class A carbapenemase producers. However, the global expansion of MBL producers now threatens the utility of these agents. In this study, we integrated clinical and genomic analyses of CZA-R-*Ec* and found that CZA-R was predominantly driven by *bla*_NDM-5_, whereas reduced activity of the ATM/CZA combination as well as AZA was largely associated with carriage of *bla*_CMY-42_ and related variants.

Because many NDM-producing isolates come from non-sterile sites (e.g., urine, sputum), clinical response can be hard to attribute to antimicrobial therapy. Our cohort’s high rate of bacteremia in profoundly immunocompromised patients offered a robust study frame to evaluate antimicrobial efficacy and link outcomes with genomics and detailed susceptibility profiles. We found that patients with CZA-R-*Ec* bacteremia carrying *bla*_NDM-5_ generally responded well to ATM/CZA and that these outcomes aligned with *in vitro* results by reference broth microdilution and gradient cross-strip testing. These findings are consistent with recent reports demonstrating strong ATM/CZA activity against NDM-producing bloodstream infections although one study was dominated by *Klebsiella pneumoniae* (12) and another included only four CZA-R-*Ec* treated with ATM/CZA monotherapy (11).

A second key finding was the clear clinical and genomic distinction between ATM/CZA-S and ATM/CZA-NS phenotypes. ATM/CZA-S isolates typically co-carried *bla*_NDM_ with *bla*_CTX-M-15_/*bla*_OXA-1_ in PBP3 YRI(K/N) insertion backgrounds and occurred in patients with less carbapenem exposure and no recent *E. coli* infection. In contrast, ATM/CZA-NS isolates tended to be isolated from patients with recent receipt of carbapenems and prior *E. coli* infection and were enriched for *bla*_CMY-42_ variants in PBP3 YRI(K/N) insertion backgrounds with frequent predicted OmpC/OmpF loss-of-function mutations. These findings are consistent with reports in *E. coli* where CMY-42, PBP3 insertions, and porin defects act together (i.e., through increased β-lactam hydrolysis, reduced target engagement, and lowered periplasmic drug levels) to reduce AZA susceptibility (20, 34–38). Clinically, our data suggest that in CMY-42/PBP3-insertion backgrounds, prior carbapenem exposure alone may select for CZA (and AZA) non-susceptibility, even without prior CZA or ATM use. Importantly, our data also suggest that CMY-42 severely impacts the utility of ATM/CZA and AZA in NDM-producing isolates with PBP3 insertions that would otherwise be susceptible to these agents, limiting therapeutic options for clinicians. As AZA becomes more widely available in the USA, the strong concordance we observed between ATM/CZA and AZA MICs supports using caution when empirically choosing AZA for CZA-R strains with this clinical and/or genomic risk profile. Additionally, our strong MIC concordance for ATM/CZA and AZA, including via ATM/CZA qualitative ETEST-based strip cross method, is a practical finding for clinical microbiology labs, lending confidence to use the ATM/CZA cross-strip approach (31, 39) as a surrogate for determining AZA susceptibility until non-research use only (RUO) commercial testing is widely available and validated.

Our long-read WGS resolved the genetic context of β-lactamase gene loci, allowing us to clearly differentiate *bla*_NDM_- and *bla*_CMY_-harboring plasmids. Our findings were consistent with other studies that detected *bla*_NDM-5_ in multireplicon IncF and to a lesser degree IncX3 genomic contexts in disparate geographical locales (24, 40, 41). In our study, 82% of CZA-R-*Ec* carried *bla*_NDM-5_, typically on multireplicon hybrid F-type plasmids, in particular, one particular subcommunity we designated F-Type Group 1 that was commonly detected in ST167 isolates collected in the US and Europe. Our plasmids frequently carried *bla*_NDM-5_ within IS*26*-mediated PCTNs, consistent with mechanisms that both amplify *bla*_NDM-5_ copy number, which is associated with increased CZA and ATM/CZA MICs, and promote mobilization (24). Notably, *bla*_NDM-5_ plasmids were found almost exclusively in strains with PBP3 insertions, echoing global observations that the majority of *E. coli* with PBP3 insertions harbor *bla*_NDM_, usually *bla*_NDM-5_ (42). In our cohort, the *bla*_CMY_ gene was with the exception of one isolate found in conserved IncI-γ/K1 plasmid backgrounds with increased plasmid copy number inferred as likely mechanism for increase in *bla*_CMY_ gene dosage as inferred by long-read pileup coverage depths. Previous studies have confirmed how increased CMY-2 activity in conjunction with reduced outer membrane porin expression can lead to progressive carbapenem resistance (43, 44). Additionally, studies have identified unique nucleotide substitutions in Inc antisense RNA structures that have been associated with increased copy numbers of IncI-γ plasmids harboring *bla*_CMY-2_ resulting in increased MICs of meropenem and imipenem (44) as well as ceftazidime and aztreonam (45), respectively. IncI complex plasmids have been shown to be a common vector for *bla*_CMY-2_ transmission (46); however, recent studies have shown that *bla*_CMY-2_ variants that develop inhibitor resistance associated with p.N346 mutations have also been associated with this plasmid type (25, 28, 47). Future studies should expand to see if increased plasmid copy number is associated with evolution of new *bla*_CMY_ variants and progressive β-lactam resistance.

Although PBP3 insertions in an NDM-5 background increase MICs to ATM/CZA and AZA (20), we observed that ATM/CZA retained both *in vitro* activity and favorable clinical response in strains with PBP3 insertions provided *bla*_CMY-42_ like variants were absent. Because CMY variants are often missing from routine AMR gene panels, these findings support confirmatory phenotypic testing of ATM/CZA or AZA activity for NDM-positive cases before finalizing therapy. Our single-center, observational design and modest sample size are limitations, but integrating detailed clinical data with long-read genomics allowed clear separation of ATM/CZA-NS and ATM/CZA-S determinants. As NDM-5 continues to expand globally (14), larger, multi-center studies are needed to define optimal empiric strategies and to determine how antimicrobial exposure, plasmid context, and mobile elements shape treatment response and the emergence of ATM/CZA or AZA non-susceptibility. Our findings also highlight *bla*_CMY-42_ and related variants, whether or not *bla*_NDM_ is present, as priority targets for continued surveillance, mechanistic study, and directed therapy development.

## MATERIALS AND METHODS

### Study design

CZA-R-*Ec* isolation that occurred at our institution from 2017-01-01 to 2024-11-30 was abstracted from Epic using a custom workbench reporting tool. Clinical data and available susceptibility testing were abstracted from Epic via chart review. The MDACC IRB panel designated this study exempt from review (OHRP IRB Registration Number: IRB00000121). Recurrent CZA-R-*Ec* is defined as one or more episodes of CZA-R-*Ec* infection or colonization occurring at least 14 days following the initial CZA-R-*Ec* infection or colonization isolate while maintaining CZA-R phenotype with infection defined using standard CDC criteria (48). Prior *E. coli* infection was considered any *E. coli* collected within 100 days from the isolation of first-available CZA-R-*Ec*. Prior carbapenem exposure was defined as one or more days of either ertapenem, meropenem, and/or imipenem therapy within 100 days prior from the index CZA-R-*Ec* isolation date. Prior cephalosporin exposure was defined as one or more days of third/fourth generation cephalosporin therapy (e.g*.*, ceftriaxone, cefepime, and/or ceftazidime) within 100 days prior to the index CZA-R-*Ec* isolation date. Exposure was counted as days of therapy (DOT) for each antibiotic which is a National Healthcare Safety Network (NHSN) metric for tracking antimicrobial use (49). Time to effective therapy was defined as the period between blood draw and administration of antibiotics with *in vitro* susceptibility. Clinical response was defined as resolution of signs and symptoms of infection (e.g*.*, fever, hypotension, tachycardia). The duration of therapy was defined as the period of administration of appropriate antibiotic treatment with two patients excluded that died during treatment.

### Antimicrobial susceptibility testing

As part of routine clinical care, antimicrobial susceptibility testing was performed in the clinical microbiology laboratory using the commercial automated Vitek 2 (bioMerieux, Marcy-l'Étoile, France) with additional susceptibility testing performed using gradient strip testing when appropriate based on Vitek 2 limitations. All ATM/CZA testing was performed using MTS (Liofilchem, Italy). Phenotypic carbapenemase testing performed using the Neo-Rapid CARB Kit (Rosco Diagnostica, Albertslund, Denmark) or RAPIDEC CARBA NP Assay (bioMérieux, Marcy-l'Étoile, France). As part of our study, reference broth microdilution was performed on all isolates per CLSI M07 and M100 guidelines using in-house prepared 96-well panels with doubling dilutions of the antimicrobial agent ranging from 0.03 to 256 µg/mL (31, 39). For CZA, AZA, and ATM/CZA, the avibactam was held constant at 4 µg/mL. In the ATM/CZA combination, the ceftazidime was held constant at 8 µg/mL. Antibiotic powders were purchased from MedChemExpress (Princeton, NJ, USA). *E. coli* ATCC 25922 (susceptible to all agents), *K. pneumoniae* BAA-2146 (New Delhi metallo-β-lactamase [NDM] producer, non-susceptible to ATM and CZA individually but susceptible to the ATM–CZA combination), and *E. coli* AR348 obtained from the AR Bank (non-susceptible to ATM, CZA, and ATM–CZA) were included as QC strains. Additionally, for this study, gradient cross-strip testing was performed for ATM/CZA using CZA MIC test strips (Liofilchem, Italy) and ATM ETEST strips (bioMérieux, Marcy-l'Étoile, France) using our previously established and validated method (39). CLSI AZA breakpoints were used to interpret results from all methods. For the analysis of associations between phenotypic susceptibility, genomic context, and resistance determinants, isolates with AZA or ATM/CZA MIC values ≤4 µg/mL were classified as susceptible (ATM/CZA-S), while isolates with MIC values >4 µg/mL were classified as nonsusceptible (ATM/CZA-NS) (30).

### Oxford nanopore technologies long-read sequencing

There was a total of 12 CZA-R-*Ec* isolates that had existing ONT data from previous projects (25, 29, 50). An additional 22 available first occurrent CZA-R-*Ec* isolates and 7 recurrent CZA-R-*Ec* isolates that were saved in our −80°C stocks were subject to ONT sequencing. Two isolates (MB14947 and CR0005) were first recurrent CZA-R-*Ec* isolates in which we did not have the index CZA-R-*Ec* isolate available for sequencing. Genomic DNA was extracted using the Sigma-Aldrich GenElute Bacterial Genomic DNA Kit per manufacturer’s instructions. The gDNA was checked for concentration and quality using the Thermo Fisher Scientific Qubit 1X dsDNA Broad Range Assay kit and Agilent Genomic DNA ScreenTape Analysis kit. ONT Library prep was performed using the ONT Rapid Barcoding Kit 96 V14 (SQK-RBK114.96) and R10.4.1 flowcells on a MinION Mk1D sequencing device. Basecalling was performed using a custom python script (run_dorado.py: https://github.com/wshropshire/misc_scripts) on the MDACC Seadragon high performance computing (HPC) cluster with the GPU enabled Dorado (v0.8.2) basecaller using a super accurate model (dna_r10.4.1_e8.2_400bps_sup@v5.0.0). Assemblies were created using a Flye assembler workflow (https://github.com/wshropshire/flyest) and manually checked and corrected for errors (51). The DNA Variant Identification using ONT (dviont-v0.2.1), which incorporates clair3 (v1.1.2) (52) variant calling using the r1041_e82_400bps_sup_v430_bacteria_finetuned model, was utilized to identify potential errors (https://github.com/wshropshire/dviont). CheckM2-v1.1.0 was used for assembly quality control with all assemblies having 100% completeness and <1% contamination (53).

### Phylogenomics

Thirty-four first available CZA-R-*Ec* isolates were selected to determine population structure of our cohort. Mash distances and FastANI were run with complete assemblies to determine which isolate had the lowest mean genetic distance from the total cohort to use as a reference for core genome alignment (reference = CR0299) (54, 55). Parsnp (v2.1.1) was used to perform the core genome alignment with the muscle aligner and recombination filter parameters (56). The core SNP alignment file was then used to infer a maximum-likelihood phylogeny using iqtree2 (v2.3.6) with ModelFinder (TVM+F + ASC+R7), ultrafast bootstrap approximation (*n* = 1,000), and SH-like aLRT branch support testing (*n* = 1,000) options (57, 58). Phylogenetic tree with metadata heatmap visualization was implemented using the R ‘ggtree’ package (v.3.10.1) (59). The core SNP alignment file generated from Parsnp was used to create a pairwise SNP distance matrix using snp-dists (v0.8.2) (https://github.com/tseemann/snp-dists). Subsequently, we constructed a minimum spanning tree (MST) of index ESC-R-*Ec* isolates, clustered using a SNP distance threshold of 15 based off previous literature (60, 61), and visualized with GraphSNP (v1.1) (62).

### blaNDM and blaCMY harboring plasmid cluster analysis

We extracted *bla*_CMY_ and *bla*_NDM-_5/*bla*NDM_-1_ positive sequences from NCBI (Accessed 2025-10) by using the Pathogen Detection Isolate Browser function to pull *E. coli* assemblies with (i) complete Amrfinder hits for the aforementioned genes, (ii) 4–6 Mb in total genome length, and (iii) less than 15 contigs. We used NCBI data sets command-line interface (CLI) to retrieve assemblies and accompanying metadata, respectively, utilizing RefSeq accession numbers pulled from Pathogen Detection Isolate Browser (https://www.ncbi.nlm.nih.gov/pathogens/isolates). Quality control was performed for all sequences using checkM2 where all assemblies had less than 5% contamination and greater than 99% completeness (53). We analyzed all extracted plasmid sequences (contigs less than 300 kb) using the mob_typer CLI function from the mob-suite toolkit (v3.1.9) and applied quality control by excluding sequences that lacked evidence of primary clustering with plasmids in our database (63). We finally clustered plasmids with Pling-v2.0.0, which first computes containment distances of plasmids and “integerises” sequences and then calculates “Double Cut and Join-Indel” (DCJ–Indel) distances and builds a plasmid network on which communities/subcommunities are called capturing both sequence variation in addition to structural rearrangements (64). Pairwise DCJ–Indel distances from Pling (all_plasmids_distances.tsv) were converted into weighted edges to construct an undirected plasmid similarity network in *R* (igraph-v2.1.4; https://r.igraph.org/). Node metadata (replicon type, sequence type, resistance genes, and collection features) were overlaid, and network visualization used a Fruchterman–Reingold layout with convex hulls outlining Pling-defined communities (ggraph-v2.2.2/ggforce-v0.4.2). The *bla*_NDM-5_ and *bla*_CMY_ plasmids from our cohort were compared all-v-all using blastn (length limit = 1,000 bp). Pairwise plasmid distances were then estimated using mash-v2.3 (54) (k-mer size 21; sketch size 1,000), and this dissimilarity matrix was used to construct a bionj tree (65). Megablast was performed on IncF Group 1 and IncI-γ/K1 Group1 plasmids, respectively, to calculate weighted mean % identity and coverage. Plasmids were then visualized using the R genoPlotR (v0.8.11) package (66).

### Additional computational genomic analysis

AMR-STRUCT (v0.1.0) was used as a wrapper of AMRFinderPlus (v.4.0.19) with the 2025-07-16.1 database to (i) identify AMR genes; (ii) perform minimap2 alignments on chromosome and plasmids (i.e., contigs >=1 Mb and < 1 Mb, respectively); and (iii) perform sliding-window median coverage depth calculations with chromosome/plasmid and identify which AMR genes were likely amplified (i.e., >1.5′ normalized coverage depth ratios) and in what context (i.e*.*, plasmid, MGE) (https://github.com/wshropshire/AMR-STRUCT). MGE predicted amplifications were confirmed by qualitatively determining if increased mapping at boundaries of predicted MGE using bam files and visualized on IGV (v.2.19.1). We have previously validated our bioinformatic approach to estimate gene copy number using qPCR analysis (50, 67). Mutations in outer membrane porin as well as penicillin binding protein encoding genes were detected using the Mutation Identification in Sequences and Frameshift/Indel Tracking (misfit-v0.0.1) with completed assemblies as input (https://github.com/wshropshire/misfit). An ONT long-read variant calling tool, the DNA Variant Identification using ONT (dviont-v0.2.1), which serves as a clair3 wrapper tool (52), was used to determine pairwise SNP distances and annotate mutations in serial isolates (https://github.com/wshropshire/dviont).

### Statistics

All statistics were computed on R-v4.5.1. Fisher’s exact test was used to test statistical significance of categorical variables, whereas Wilcoxon rank-sum tests were used to test statistically significant differences of continuous variables. *P*-values were adjusted using the Benjamini-Hochberg procedure to control the false discovery rate in cases where multiple comparisons (e.g., antibiotic usage, AMR gene/feature) were made.

## Data Availability

Genomic data and assemblies from prior publications are available under NCBI BioProjects PRJNA836696, PRJNA924946, and PRJNA1150149. Data generated for this study have been deposited under PRJNA1308153 with data details and BioSample accession numbers provided in Table S1. All other data produced in the present study are available upon reasonable request to the authors.

## SUPPLEMENTAL MATERIAL

Supplemental figures 1 aac.01860-25-s0001.docx Fig. S1 to S3.

Supplemental figures 2 aac.01860-25-s0002.docx Fig. S4 to S6.

Supplemental tables aac.01860-25-s0003.xlsx Tables S1 to S8.

ASM does not own the copyrights to Supplemental Material that may be linked to, or accessed through, an article. The authors have granted ASM a non-exclusive, world-wide license to publish the Supplemental Material files. Please contact the corresponding author directly for reuse.
