# Supplementary material for: Clinical and genomic determinants associated with emergent ceftazidime–avibactam plus aztreonam non-susceptibility in ceftazidime-avibactam resistant Escherichia coli
Source: Antimicrob Agents Chemother. 2026 Mar 23;70(5):e01860-25. doi: 10.1128/aac.01860-25 (PMC13148019; doi:10.1128/aac.01860-25)
Supplement: Supplemental figures 1 — Fig. S1 to S3. [file aac.01860-25-s0001.docx]

**SUPPLEMENTAL FIGURES**

**
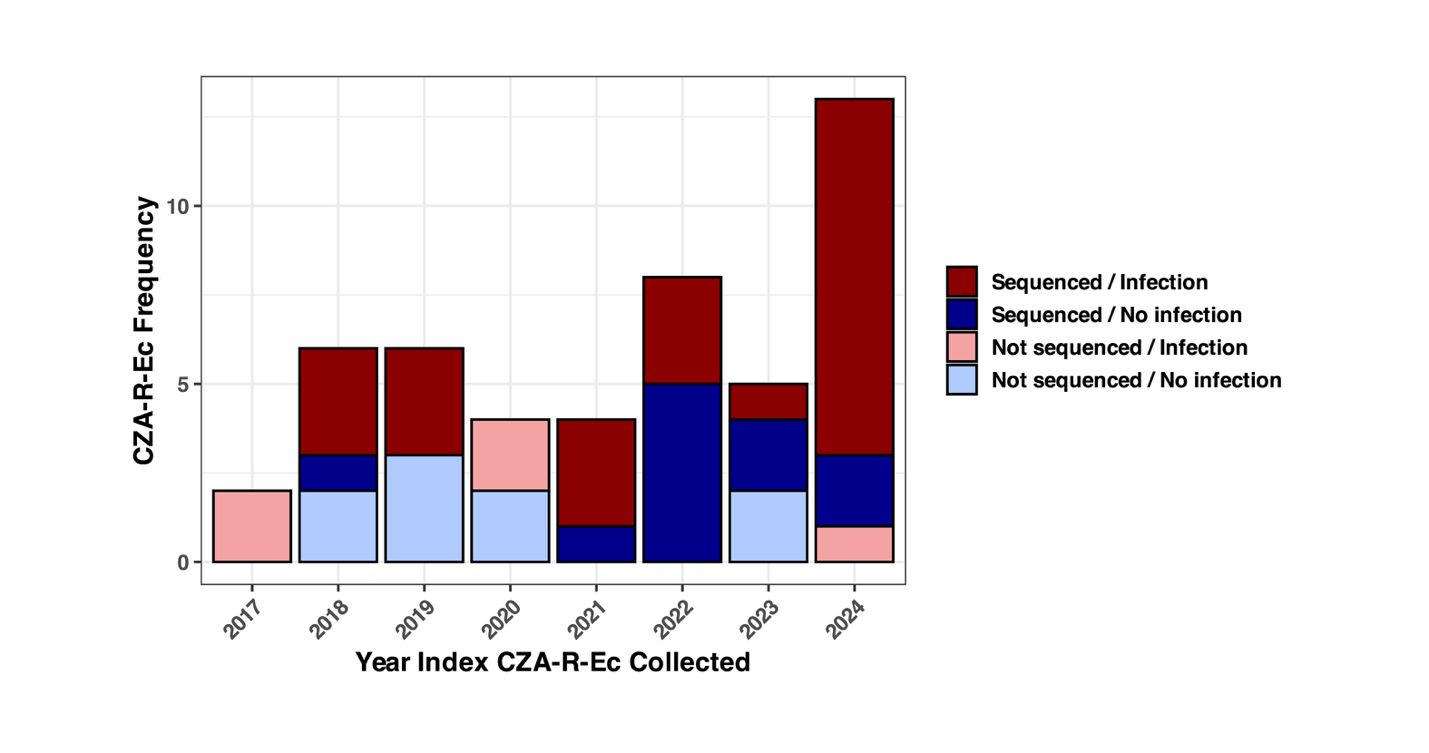
**

**Fig S1** Frequency of CZA-R-Ec isolates collected (n=48) over time (2017-2024) stratified by sequence status (sequenced=dark; not sequenced=light shading) and infection status (red=infection; blue=colonization).


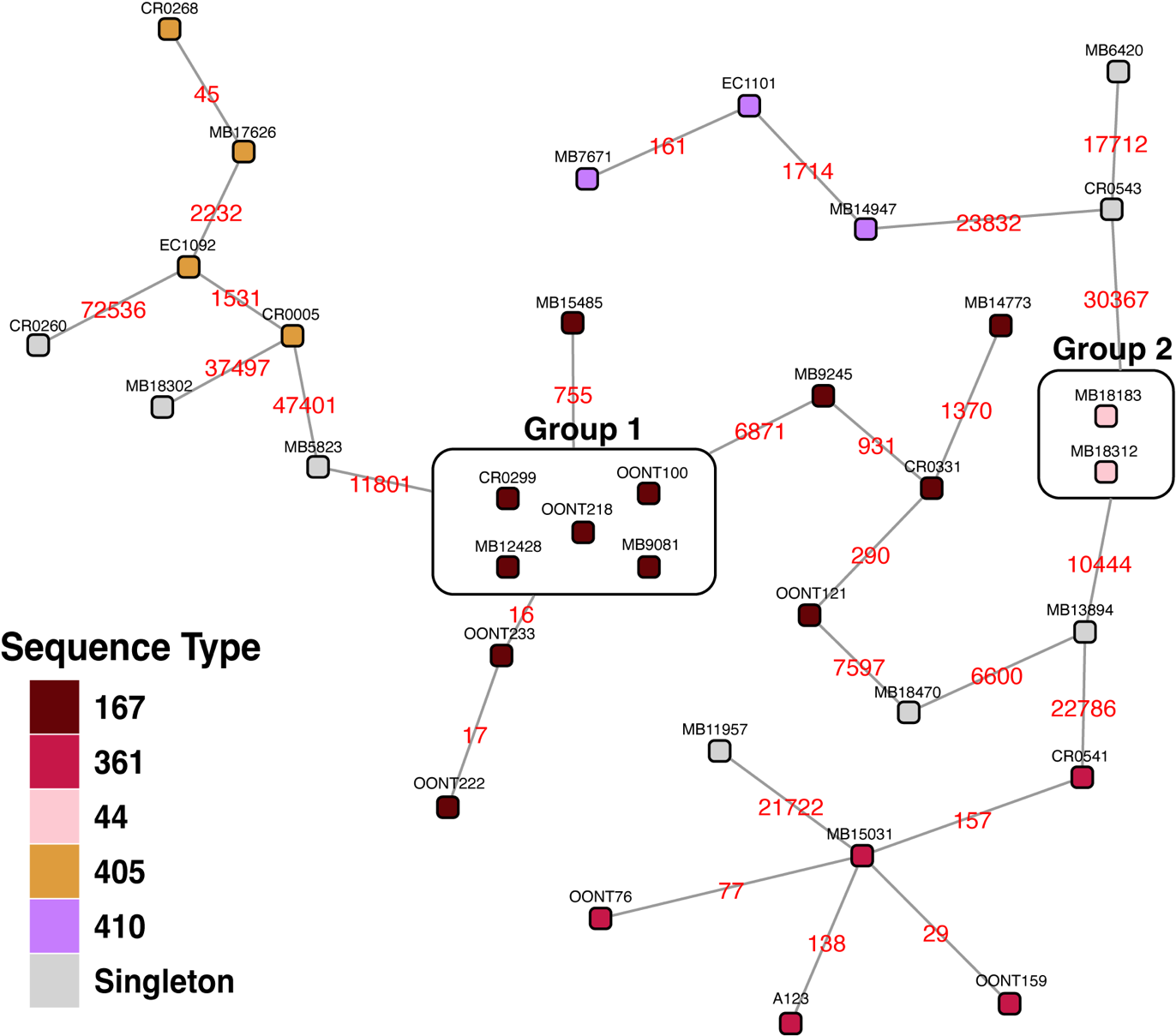


**Fig S2** Minimum spanning tree with nodes (isolates) connected by edges (pairwise SNP distances) with red text indicating pairwise SNP distance. Nodes are colored by sequence type with Group 1 ST167 and Group 2 ST44 isolates (<15 pairwise SNPs) circled and labelled respectively.

**
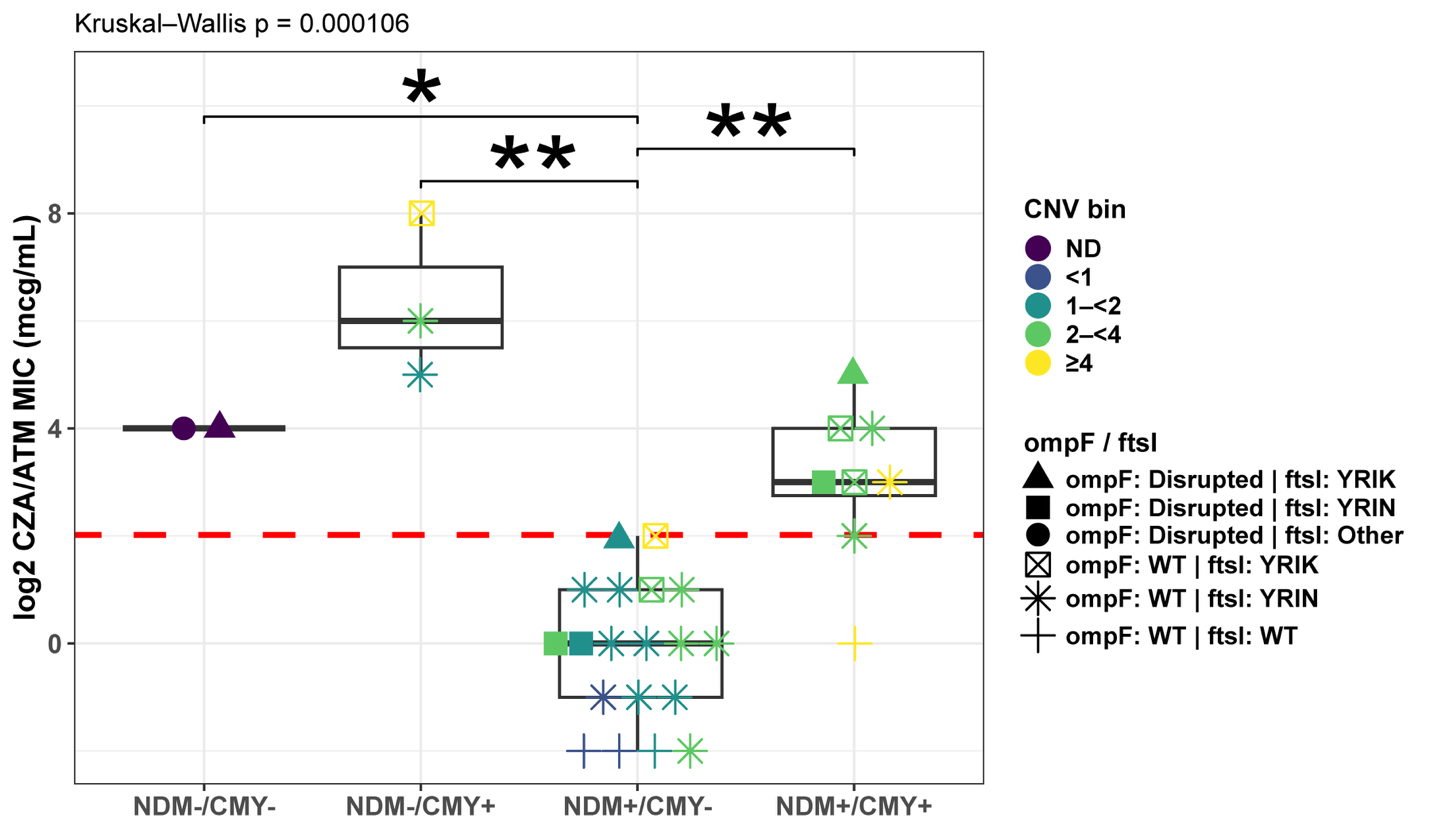
**

**Fig S3** Log2 converted ceftazidime-avibactam plus aztreonam (CZA/ATM) minimum inhibitory concentrations (MIC) stratified by *bla*_NDM_ and/or *bla*_CMY_ carriage. Red dotted horizontal line indicates CZA/ATM breakpoint (4 μg/mL). Pairwise Mann-Whitney U adjusted *P*-value designations: *P<0.05; **P<0.01. *bla*_NDM_ and/or *bla*_CMY_ copy number variant (CNV) estimation is additive in the NDM+/CMY+ group.
