# Supplementary material for: Clinical and genomic determinants associated with emergent ceftazidime–avibactam plus aztreonam non-susceptibility in ceftazidime-avibactam resistant Escherichia coli
Source: Antimicrob Agents Chemother. 2026 Mar 23;70(5):e01860-25. doi: 10.1128/aac.01860-25 (PMC13148019; doi:10.1128/aac.01860-25)
Supplement: Supplemental figures 2 — Fig. S4 to S6. [file aac.01860-25-s0002.docx]

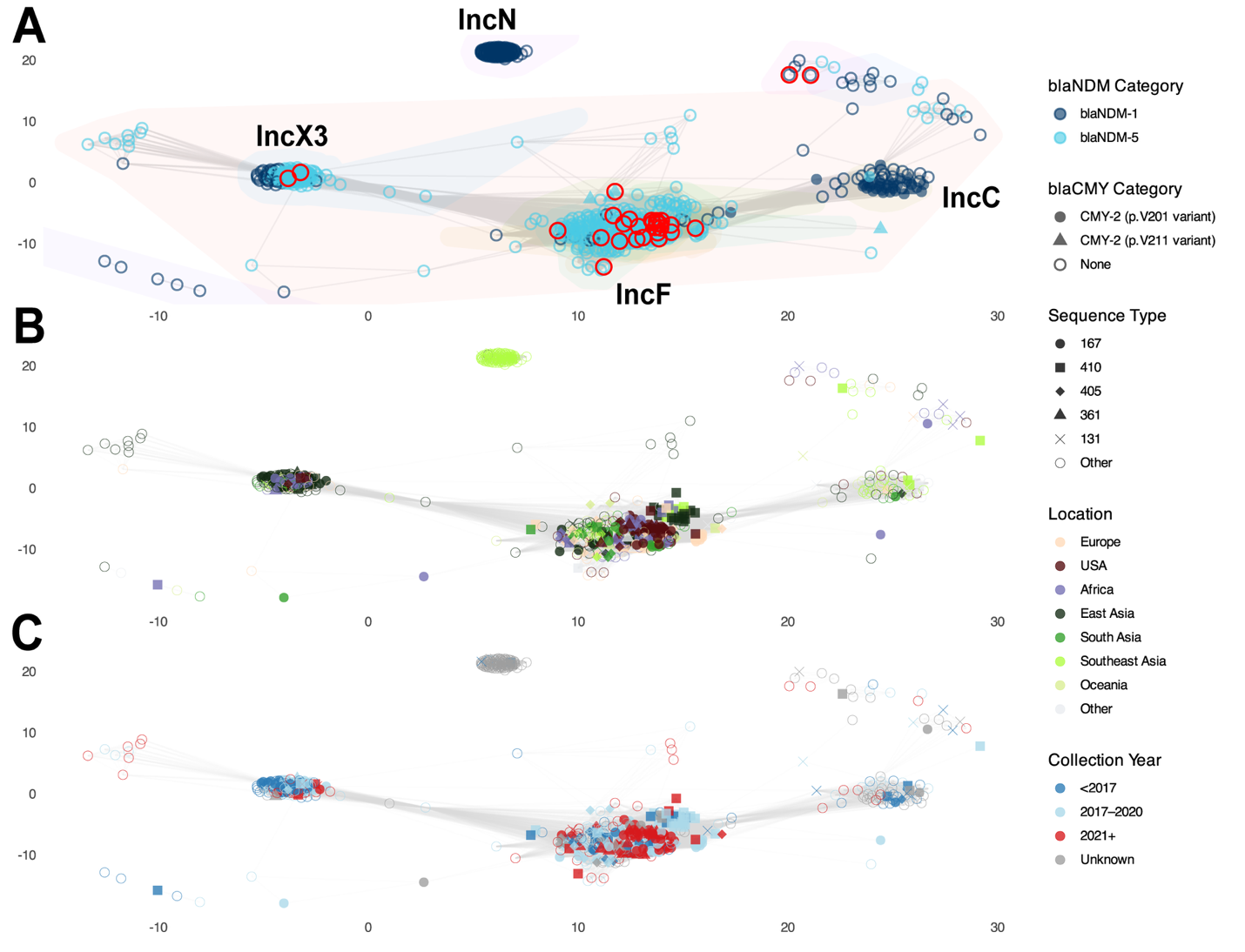


**Fig S4** NDM plasmid network analysis stratified by **(A)** *bla*_NDM_ variant carriage and *bla*_CMY_ variant carriage; **(B)** sequence type and location collected; **(C)** sequence type and collection year. Plasmid groups are labelled as well as red circles identifying this study plasmid cohort in panel **A**.


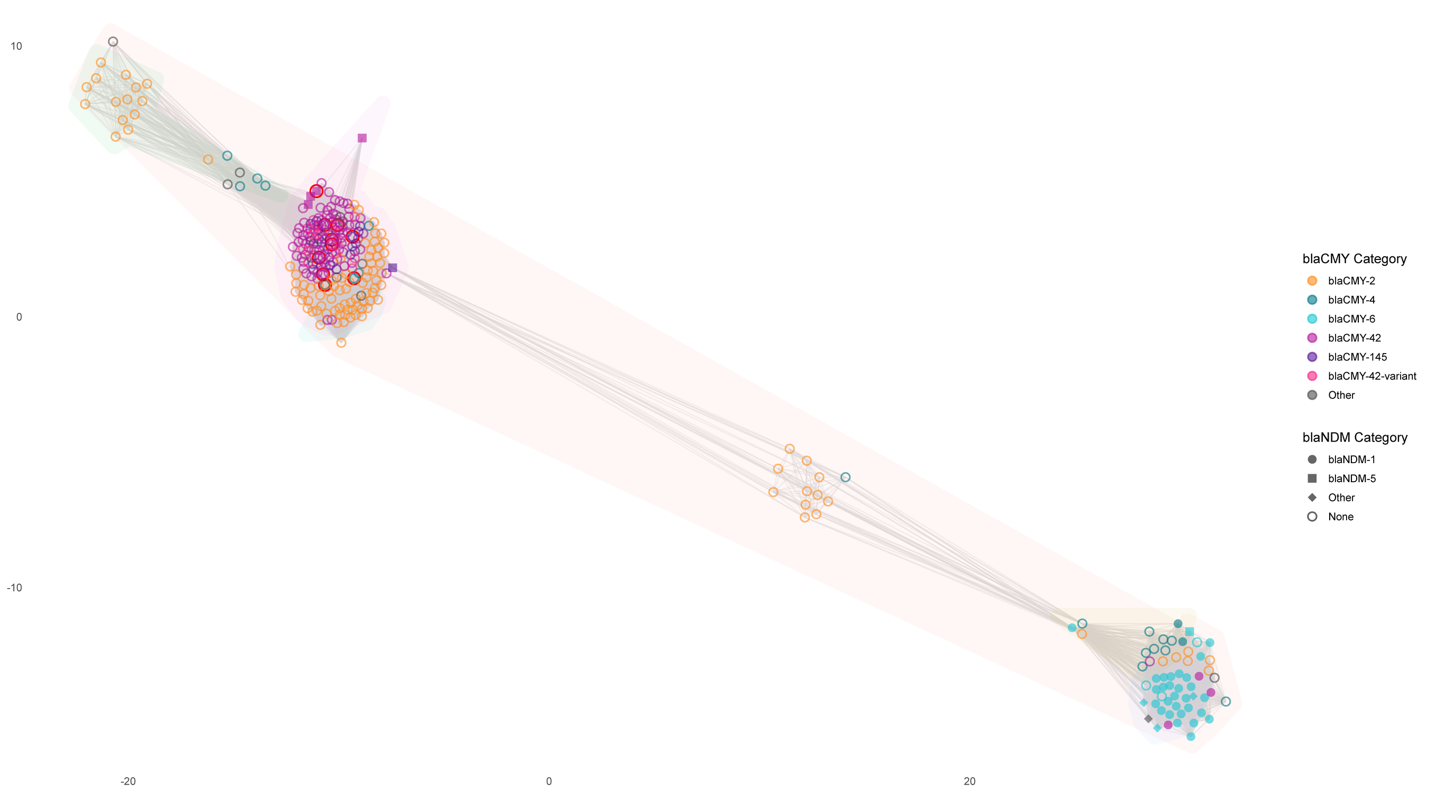


**Fig S5** CMY plasmid network analysis stratified by *bla*_NDM_ variant carriage and *bla*_CMY_ variant carriage. Red circles identify this study plasmid cohort.


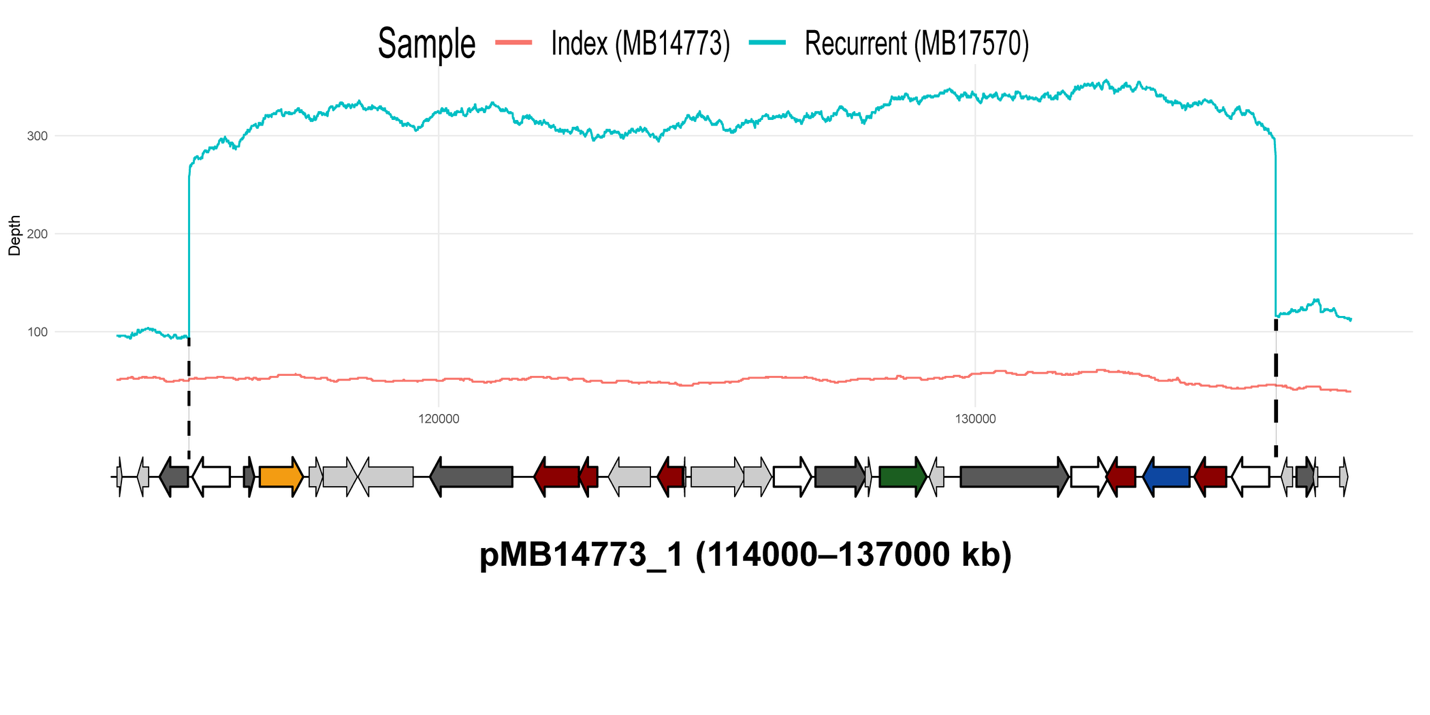


**Fig S6** Read mapping per base coverage depth of pMB14773_1 region (114000 to 137000 kb) for index (red line) vs recurrent (blue line) isolate indicating PCTN-mediated amplification of IS*26* (white arrows) mediated PCTN harboring *bla*_NDM-5_ (orange arrow), *bla*_CTX-M-15_ (green arrow), *bla*_OXA-1_ (blue arrow), and other AMR genes (dark red arrows). Dark gray arrows indicate other transposases present. Dotted vertical black line indicates the boundaries of IS*26* amplification demarcated by the right inverted repeat (IRR) and left inverted repeat (IRL) of cis oriented IS*26* transposase genes reading left to right.
